# Self-Care, Hope, and Life Meaning as Predictors of Psychological Resilience in Informal Caregivers of Hemodialysis Patients: A Systematic Review

**DOI:** 10.7759/cureus.100630

**Published:** 2026-01-02

**Authors:** Charikleia Vidinioti, Zoe Konstanti, Mary Gouva, Michael Kourakos

**Affiliations:** 1 Research Laboratory Psychology of Patients, Families and Health Professionals, Department of Nursing, School of Health Sciences, University of Ioannina, Ioannina, GRC

**Keywords:** hemodialysis, informal caregivers, life meaning, psychological resilience, self-care, spirituality

## Abstract

Background: The physical, emotional, and psychological difficulties that informal caregivers of hemodialysis patients commonly encounter can jeopardize their health. Spirituality, life purpose, and self-care may be protective variables that increase psychological resilience in this population, according to recent research.

Objective: In this systematic review, quantitative data on the predictive power of spirituality, life purpose, and self-care in predicting psychological resilience in informal caregivers of hemodialysis patients is critically assessed and synthesized.

Methods: A comprehensive search of PubMed, PsycINFO, Scopus, Web of Science, and CINAHL was conducted for studies published between 2010 and 2025. We included quantitative studies that evaluated spirituality, self-care, life meaning, and resilience in informal caregivers of adult hemodialysis patients. Eighteen studies satisfied the inclusion criteria after 65 full-text articles were assessed, 47 entries were filtered by title and abstract, and duplicates were eliminated. Measurement instruments, research features, and statistical correlations were the main topics of data extraction. To assess the quality of the methodology, the Joanna Briggs Institute Critical Appraisal Checklists were used. A narrative synthesis was performed since study designs and measures varied widely.

Results: Higher levels of life purpose, spiritual well-being, self-efficacy, hope, and self-care were consistently linked to stronger psychological resilience and less caregiver strain across the 18 investigations. Improved coping mechanisms and reduced stress were observed among caregivers with higher spiritual well-being, a clearer sense of life purpose, or stronger self-care routines. Additionally, resilience mitigated the effects of caregiver stress, which modulated psychological outcomes.

Conclusion: Spirituality, life purpose, and self-care all have a significant impact on the psychological resilience of informal caregivers of hemodialysis patients. The well-being of caregivers and their ability to provide long-term care may be improved by interventions that focus on these variables.

## Introduction and background

Chronic kidney disease (CKD) requiring hemodialysis represents a major global health burden, with increasing prevalence and substantial impacts on patients' physical and emotional well-being. Hemodialysis is a life-sustaining procedure, but it is also very taxing, including frequent medical monitoring, dietary restrictions, and rigorous adherence to treatment plans. Patients typically depend heavily on unpaid caregivers who provide continuous logistical, emotional, and physical support. According to Cohen et al., these caregivers are frequently close friends or family members [1].

Hemodialysis caregiving represents a global public health concern, particularly in low- and middle-income countries where healthcare systems rely heavily on informal family support [2]. Cultural norms strongly influence caregiving roles, expectations, and coping strategies, with family-centered care being more prominent in Asia and the Middle East, while greater formal support structures exist in Western contexts. These cultural differences may shape how caregivers experience burden, meaning-making, spirituality, and resilience [3].

Caregiving can expose informal caregivers to considerable stress, anxiety, and depressive symptoms, potentially compromising their health, while simultaneously being crucial to patient survival and quality of life [4]. The prolonged and unpredictable nature of dialysis-related caregiving places caregivers at heightened risk for psychological strain and exhaustion. These challenges underscore the importance of understanding factors that support resilience in this population. Informal caregivers of hemodialysis patients frequently report high levels of psychological distress, role strain, and disruptions to employment, social functioning, and physical health, reflecting the sustained and intensive nature of caregiving responsibilities.

Psychological resilience, defined as the ability to adapt constructively to stress and adversity, has been identified as a key protective factor for caregiver well-being [5]. Resilient caregivers are better able to maintain psychological balance, solve problems effectively, and preserve their quality of life. Resilience, however, is shaped by a combination of personal, contextual, and social resources and does not develop in isolation. Across the studies included in this review, resilience is variably conceptualized as a relatively stable personal capacity, as an outcome influenced by psychosocial and spiritual factors, or as a mediating or moderating mechanism linking caregiving stressors to psychological outcomes.

Among the personal resources associated with resilience, self-care, hope, and life meaning have gained increasing recognition. Self-care, which encompasses intentional actions to preserve physical, emotional, and mental health, enables caregivers to manage stress and reduce burnout [6]. Hope has been demonstrated to support motivation, optimism, and coping capacity in the context of chronic stress [7]. Similarly, a strong sense of life meaning, defined as the perception that one’s experiences are purposeful and significant, can mitigate the impact of prolonged caregiving stress and facilitate adaptive coping strategies [8].

Although prior research has examined caregiver burden and quality of life in CKD, there is limited quantitative synthesis focusing specifically on personal and existential resources, such as self-care, hope, meaning in life, and spirituality, as predictors of psychological resilience among informal caregivers of hemodialysis patients.

This systematic review critically examines and synthesizes quantitative studies on self-care, life meaning, and spirituality as predictors of psychological resilience among informal caregivers of hemodialysis patients.

## Review

Methods

Search Strategy

This systematic review was conducted in accordance with the PRISMA 2020 guidelines. A comprehensive literature search was performed in PubMed, MEDLINE, and Scopus to identify studies published between 2010 and 2025. Medical Subject Headings (MeSH) and relevant free-text terms were combined using Boolean operators. The search strategy included the following terms: (“Caregivers”[MeSH] OR caregiver* OR “informal caregiver*” OR “family caregiver*”) AND (“Renal Dialysis”[MeSH] OR hemodialysis OR dialysis OR “end-stage renal disease” OR ESRD) AND (“Resilience, Psychological”[MeSH] OR resilience OR “psychological resilience” OR coping OR hope) AND (“Self-Care”[MeSH] OR “self-management” OR “meaning in life” OR “purpose in life” OR “life meaning”).

Database-specific adaptations were applied to optimize sensitivity and precision. In addition, reference lists of all included studies and relevant reviews were manually screened to identify additional eligible articles.

Eligibility Criteria

Studies were eligible if they: (1) involved informal or family caregivers of adult patients undergoing hemodialysis; (2) assessed psychological resilience or related constructs such as coping, hope, meaning in life, or spiritual well-being; and (3) employed an empirical study design (cross-sectional, cohort, interventional, or qualitative). Only peer-reviewed articles published in English between 2010 and 2025 were included. 

Studies were excluded if they focused on caregivers of pediatric patients, peritoneal dialysis patients, kidney transplant recipients, or professional caregivers. Reviews, conference abstracts, commentaries, and study protocols were excluded. Studies that focused exclusively on caregiver burden, quality of life, or sleep outcomes without assessing resilience or meaning-based constructs were also excluded, as were non-English publications.

Study Selection

**Figure 1:**
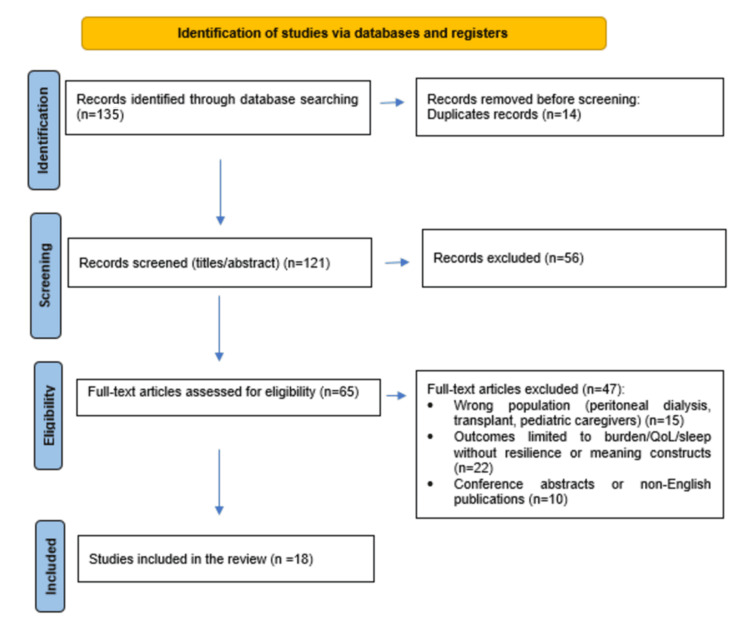
PRISMA 2020 flow diagram illustrating the identification, screening, eligibility assessment, and inclusion of studies, including reasons for full-text exclusions. PRISMA: Preferred Reporting Items for Systematic Reviews and Meta-Analyses.

The database search identified 135 records. After removal of 14 duplicates, 121 titles and abstracts were screened, resulting in the exclusion of 56 records that did not meet the eligibility criteria. The full texts of 65 articles were subsequently assessed for eligibility. Of these, 47 were excluded for the following reasons: incorrect study population (peritoneal dialysis, transplant, or pediatric caregivers; n = 15), outcomes limited to caregiver burden or quality of life without resilience- or meaning-related measures (n = 22), or publication type or language restrictions (conference abstracts or non-English publications; n = 10). Titles and abstracts were screened independently by two reviewers, followed by full-text assessment for eligibility, with disagreements resolved by consensus. A total of 18 studies met the inclusion criteria and were included in the final synthesis. Figure 1 presents the PRISMA 2020 flow diagram of the study selection process.

Data Extraction

A standardized data extraction form was used to collect information from each included study, including author(s) and year of publication, country and setting, study design, sample size, caregiver characteristics (age, gender, relationship to patient), measurement instruments used to assess resilience or related constructs, and main findings, including reported statistical associations. Data extraction was performed independently by two reviewers, with discrepancies resolved through discussion and consensus.

Quality Appraisal

The methodological quality of included studies was assessed using the Joanna Briggs Institute (JBI) Critical Appraisal Checklists appropriate to each study design. Assessment criteria included sample representativeness, validity and reliability of measurement instruments, and appropriateness of statistical analyses. Methodological quality was not used as an exclusion criterion but was considered when interpreting and synthesizing findings, with common sources of bias summarized narratively rather than reported at the individual study level [9].

Data Synthesis

Given the substantial heterogeneity in study designs, populations, outcome definitions, and measurement instruments, a narrative synthesis was conducted. Findings were summarized thematically, with emphasis on recurring patterns and differences across studies, and organized according to key predictors and outcomes of interest, including psychological resilience, coping strategies, hope, and meaning or purpose in life among informal caregivers of adult hemodialysis patients. Themes were derived inductively by grouping conceptually similar outcomes across studies and were prioritized based on frequency of reporting and consistency of associations. Owing to heterogeneity in outcome measures, scales, and statistical reporting, no quantitative pooling of effect sizes, confidence intervals, or p-values was attempted, and meta-analysis was not feasible.

Results

A total of 135 records were identified through database searches. After removal of 14 duplicates, 121 titles and abstracts were screened, resulting in the exclusion of 56 records. The full texts of 65 articles were assessed for eligibility, of which 47 were excluded for the following reasons: incorrect study population (peritoneal dialysis, transplant, or pediatric caregivers; n = 15), outcomes limited to caregiver burden or quality of life without resilience- or meaning-related outcomes (n = 22), or publication type or language restrictions (conference abstracts or non-English publications; n = 10). Ultimately, 18 studies were included in the final synthesis.

Study Characteristics and Key Findings

Across included studies, resilience was variably conceptualized as an outcome, a protective trait, or a mediating/moderating construct, reflecting differences in theoretical frameworks. Eighteen studies were included in this systematic review, comprising 13 cross-sectional, three interventional, and two qualitative studies. Sample sizes ranged from 28 to 315 caregivers, and the studies were conducted in diverse geographic regions, including Asia, the Middle East, North America, South America, and Europe. Key constructs examined included resilience, coping, hope, spiritual well-being, and meaning in life. Across studies, higher levels of resilience were consistently associated with lower caregiver burden, better coping capacity, and improved psychological outcomes (Table 1). Interventional studies suggested that structured programs, such as empowerment models, psychoeducational support groups, and stress management training, can enhance resilience, although sample sizes were generally small and follow-up periods were short. Qualitative studies provided complementary insights into the lived experiences of caregivers, highlighting the roles of hope, meaning-making, and spiritual coping in promoting resilience.

**Table 1:** Summary of included studies examining psychological resilience, self-care, hope, and life meaning among informal caregivers of hemodialysis patients. This table provides an overview of the 18 studies included in the systematic review, presenting key methodological characteristics and primary findings for each investigation. For every study, the table summarizes the author(s), publication year, country, research design, sample size, and the main outcomes related to psychological resilience, coping, hope, spiritual well-being, and meaning in life.

Author(s)	Year	Country	Design	Sample size (caregivers)	Key findings	Main outcome
Joy et al. [10]	2019	India	Cross-sectional	120	Resilience inversely related to burden; higher resilience improved coping.	Resilience
Abdullah et al. [11]	2021	India	Cross-sectional	106	Resilience buffered the psychological impact of burden; better psychological outcomes.	Resilience as moderator
Affinito and Louie [12]	2018	USA	Cross-sectional	204	Positive coping associated with lower burden and better self-rated health.	Coping and resilience
Sajadi et al. [13]	2021	Iran	Cross-sectional	250	Hope positively associated with QoL and coping capacity.	Hope, QoL, resilience
Zhang et al. [14]	2024	China	Cross-sectional	315	Family resilience mediated coping styles and caregiver burden.	Family resilience
Rafati et al. [15]	2020	Iran	Cross-sectional	220	Spiritual well-being correlated with lower burden and improved resilience.	Spiritual well-being
Akbari et al. [16]	2023	Iran	Cross-sectional	178	Fatigue negatively associated with resilience and QoL.	Resilience, fatigue, QoL
Kiani et al. [17]	2023	Iran	Interventional (RCT)	90	Empowerment model improved resilience scores significantly.	Resilience intervention
Khouban-Shargh et al. [18]	2024	Iran	Interventional (RCT)	100	Stress management training increased resilience and coping capacity.	Resilience intervention
Nuriyyatiningrum et al. [19]	2020	Indonesia	Interventional (quasi-experimental)	75	Psychoeducational support group improved resilience.	Resilience intervention
Azeez and Ambatipudi [20]	2024	India	Cross-sectional	150	Higher resilience associated with better psychological outcomes.	Resilience, QoL
Al Maqbali et al. [21]	2024	Oman	Cross-sectional	200	Resilience predicted caregiver burden and psychological health.	Resilience predictors
Hejazi et al. [22]	2021	Iran	Qualitative	30	Themes of coping and resilience identified among caregivers.	Qualitative resilience
Hejazi et al. [23]	2022	Iran	Qualitative	32	Explored resilience, hope, and meaning in caregivers’ narratives.	Qualitative resilience/hope
Hejazi et al. [24]	2024	Iran	Qualitative	28	Discussed pathways to resilience in caregiving context.	Qualitative resilience
Lim et al. [25]	2023	Singapore	Cross-sectional	260	Resilience correlated with lower burden and better psychological outcomes.	Resilience and burden
Pereira et al. [26]	2017	Brazil	Cross-sectional	110	Resilience and hope linked with better mental health.	Resilience and hope
Sousa et al. [27]	2024	Portugal	Cross-sectional	140	Purpose in life linked with adaptive coping and lower distress.	Meaning/purpose

Study-level limitations: Study-level limitations are summarized in Table 2. Common methodological constraints included the predominance of cross-sectional designs, reliance on self-reported measures, small sample sizes, and short-term follow-up in interventional studies. Cultural specificity and limited geographic diversity also emerged as recurrent limitations, indicating that the generalizability of findings may be restricted. Addressing these limitations will require longitudinal, culturally diverse, and adequately powered studies capable of advancing understanding of the predictors and mechanisms of caregiver resilience. 

**Table 2:** Summary of study-specific methodological and contextual limitations that may influence interpretation and generalizability of findings. This table summarizes the main methodological and contextual limitations identified in each included study. Limitations reported by the authors include small sample sizes, cross-sectional designs, potential self-report bias, cultural specificity, short follow-up periods in interventional research, and limited adjustment for confounders. These limitations were considered when interpreting the overall strength of the evidence.

Author(s)	Year	Key specific limitations
Joy et al. [10]	2019	Self-reported measures may introduce bias; limited assessment of social support or contextual factors
Abdullah et al. [11]	2021	Single-center study; lack of adjustment for potential confounders; cultural context may limit generalizability
Affinito and Louie [12]	2018	Coping measure not validated across diverse populations; limited demographic diversity in sample
Sajadi et al. [13]	2021	Cross-sectional design limits causal inference; single-center; possible cultural specificity of hope measure
Zhang et al. [14]	2024	Family resilience scale not standardized; single-region study; limited assessment of external stressors
Rafati et al. [15]	2020	Spiritual well-being assessed by self-report; did not account for other psychosocial variables that may influence resilience
Akbari et al. [16]	2023	Focused on short-term outcomes; fatigue measured via self-report only; potential confounding factors not controlled
Kiani et al. [17]	2023	Small sample; short follow-up limits assessment of long-term intervention effects; single-center
Khouban-Shargh et al. [18]	2024	Short follow-up; intervention may not be generalizable outside study setting; limited reporting of adherence
Nuriyyatiningrum et al. [19]	2020	Non-randomized design reduces internal validity; small sample; short-term outcomes only
Azeez and Ambatipudi [20]	2024	Single-region study; cross-sectional; limited exploration of mediating factors influencing resilience
Al Maqbali et al. [21]	2024	Cultural specificity may limit generalizability; limited control for confounding variables; cross-sectional design
Hejazi et al. [22]	2021	Small qualitative sample; findings may not generalize beyond the cultural context; potential researcher interpretation bias
Hejazi et al. [23]	2022	Single-center; subjective narrative interpretation; limited demographic diversity
Hejazi et al. [24]	2024	Small sample; focus on specific cultural context limits transferability; potential researcher bias in coding themes
Lim et al. [25]	2023	Cross-sectional; self-reported measures may introduce bias; limited assessment of long-term psychological outcomes
Pereira et al. [26]	2017	Single-site study; cross-sectional; limited exploration of confounding variables
Sousa et al. [27]	2024	Cross-sectional; limited adjustment for potential confounders; short-term assessment only

Narrative Synthesis

Across studies, higher resilience and stronger meaning in life were consistently associated with improved caregiver well-being and reduced psychological distress. Rafati et al. [15] showed that spiritual well-being predicted lower burden, while Sousa et al. [27] highlighted the role of purpose in life in supporting adaptive coping and decreasing distress.

Hope and self-efficacy emerged as additional resilience-related constructs. Evidence from prior studies suggests that various psychological factors mediate or amplify the effects of social and emotional resources. For instance, Taherkhani et al. [28] identified self-efficacy as a mediator of the relationship between social support and stress reduction, whereas Sajadi et al. [13] observed that greater hope predicted more adaptive coping and better quality of life.

The protective role of resilience against caregiving-related stress has been directly supported by studies that explicitly assessed resilience among caregivers. Joy et al. [10] and Abdullah et al. [11] found that higher resilience among caregivers was associated with improved psychological outcomes and reduced negative effects of caregiving burden. Interventional research also suggests that targeted stress management and psychoeducational programs are effective in strengthening caregiver resilience [17-19].

Collectively, these findings suggest that personal resources, such as spiritual well-being, hope, self-efficacy, and a sense of life purpose, play an important role in helping informal caregivers of adult hemodialysis patients. Enhancing these resources may help caregivers preserve mental well-being, lessen perceived stress, and adapt more effectively to the challenges of caregiving.

Discussion

Individual resources, including hope, coping, self-efficacy, spiritual well-being, and meaning in life, were the focus of this systematic review, which synthesized evidence on the psychological resilience of informal caregivers of adult hemodialysis patients. Across the 18 included studies, these personal characteristics consistently emerged as factors that support greater resilience and mitigate caregiver burden. Although hemodialysis therapy is a chronic and demanding treatment, the findings underscore the essential contribution of psychological and spiritual resources to sustaining caregiver well-being.

Interpretation of Findings

Resilience was most strongly correlated with life purpose and spiritual well-being. Adaptive functioning and stress management were more easily maintained by caregivers who had a greater sense of purpose or a connection to a higher power [15,27]. Engagement in meaning-making appears to reduce perceived strain by allowing caregivers to appraise their caregiving responsibilities more positively.

Hope and self-efficacy also emerged as key resources for promoting resilience. According to Taherkhani et al. [28], self-efficacy moderated the relationship between social support and stress reduction, but greater hope was associated with better coping skills and a higher quality of life [11]. These results are in line with theoretical frameworks that view resilience as a dynamic process driven by optimism, goal-oriented thinking, and self-belief in one's capacity for coping.

Research directly examining resilience supports its role as a protective factor against caregiver stress. Findings from Joy et al. [10] and Abdullah et al. [11] indicate that greater resilience correlates with lower stress and enhanced psychological health. Family resilience was also highlighted in some studies, demonstrating the relational dimension of adaptive coping [14].

Interventional studies demonstrated that resilience can be enhanced through structured programs, such as psychoeducational support groups, empowerment models, and stress management training [17-19]. However, these studies were small and often short-term, highlighting the need for larger, longer-term trials.

Qualitative studies enriched understanding of mechanisms underlying resilience, emphasizing the role of hope, meaning-making, and spiritual coping in caregivers' lived experiences [22-24]. These insights complement quantitative findings and suggest pathways through which interventions could be designed.

Implications for Practice

Healthcare professionals should consider interventions that strengthen caregivers' internal resources. Programs enhancing spiritual well-being, cultivating a sense of purpose, fostering hope, and improving self-efficacy may help caregivers manage stress and maintain resilience. Regular assessment of these factors, integrated into caregiver support strategies, could improve both caregiver well-being and patient care.

Limitations

Several limitations should be considered when interpreting the findings of this review. Most notably, the majority of included studies were cross-sectional, which restricts the ability to draw causal conclusions. While associations between psychological resources, such as hope, self-efficacy, and spiritual well-being, and resilience were consistently observed, it remains unclear whether these factors actively promote resilience or are a consequence of being more resilient.

The diversity of measurement tools across studies also posed challenges. Resilience, coping, hope, and spiritual well-being were assessed using a range of scales, which limited comparability and prevented quantitative synthesis of results. In addition, full database-specific search strings and domain-level, study-by-study risk-of-bias assessments were not reported, which may limit methodological transparency. The geographic concentration of studies in Asia and the Middle East also raises questions about generalizability. Cultural differences in caregiving roles, spiritual practices, and meaning-making are likely to influence resilience, yet these factors were not consistently examined.

Sample sizes in several studies, particularly interventional and qualitative studies, were relatively small, which may limit the robustness and reliability of findings. Although intervention studies suggest that resilience can be enhanced through psychoeducational programs, empowerment models, or stress management training, the number of such studies remains limited, and follow-up periods were often short. Consequently, the long-term effectiveness of these interventions remains uncertain.

Finally, potential publication and language biases may have affected the body of evidence. Studies reporting positive outcomes are more likely to be published, and non-English publications may have been underrepresented, potentially narrowing the scope of findings. Taken together, these limitations underscore the need for larger, longitudinal, and culturally diverse studies using standardized measures of resilience and related constructs, as well as well-designed intervention trials with longer-term follow-up, to better understand the factors that support caregiver resilience.

Future directions

Future research should prioritize longitudinal designs to better understand causal pathways between psychological resources and resilience among caregivers. There is also a need for more interventional studies to test strategies for enhancing self-efficacy, hope, and life purpose, with attention to evaluating their long-term effects. Cross-cultural investigations would help clarify how cultural norms and values shape the ways caregivers draw on personal and spiritual resources. Additionally, combining qualitative and quantitative approaches could provide deeper insights into the lived experiences of caregivers, enriching our understanding of resilience in real-world contexts.

## Conclusions

This systematic review demonstrates that psychological resilience among informal caregivers of adult hemodialysis patients is strongly influenced by self-efficacy, hope, spiritual well-being, and a sense of life purpose. Interventions aimed at strengthening these personal resources have the potential to enhance adaptive coping, reduce stress, and support sustained caregiving. Fostering resilience not only benefits caregivers’ psychological well-being but may also improve patient outcomes by ensuring more stable and effective informal support networks.
